# Factorization of Dual Quaternion Polynomials Without Study’s Condition

**DOI:** 10.1007/s00006-021-01123-w

**Published:** 2021-03-05

**Authors:** Johannes Siegele, Martin Pfurner, Hans-Peter Schröcker

**Affiliations:** Department of Basic Sciences in Engineering Sciences, University of Innsbruck, Techikerstr. 13, 6020 Innsbruck, Austria

**Keywords:** Factorization, Dual quaternions, Dual quaternion polynomials, Rational motion, Skew polynomial ring, Construction of mechanisms, Primary 16S36, Secondary 70B15

## Abstract

In this paper we investigate factorizations of polynomials over the ring of dual quaternions into linear factors. While earlier results assume that the norm polynomial is real (“motion polynomials”), we only require the absence of real polynomial factors in the primal part and factorizability of the norm polynomial over the dual numbers into monic quadratic factors. This obviously necessary condition is also sufficient for existence of factorizations. We present an algorithm to compute factorizations of these polynomials and use it for new constructions of mechanisms which cannot be obtained by existing factorization algorithms for motion polynomials. While they produce mechanisms with rotational or translational joints, our approach yields mechanisms consisting of “vertical Darboux joints”. They exhibit mechanical deficiencies so that we explore ways to replace them by cylindrical joints while keeping the overall mechanism sufficiently constrained.

## Introduction

Factorization of dual quaternion polynomials is a powerful tool for the systematic construction of mechanisms which can perform a given rational motion [3, 4]. In [5] it has been shown that rational motions can be parametrized by motion polynomials, which are defined as polynomials over the ring of dual quaternions with real norm polynomial (Study’s condition). Factorization algorithms for motion polynomials are presented in [3, 7]. They decompose a given polynomial into products of linear motion polynomials. The obtained factors parametrize rotations or translations. Factorizations are generically non-unique. This allows for the construction of mechanisms with multiple “legs”, each corresponding to one factorization. One leg consists of revolute or prismatic joints, obtained from linear factors in one factorization.

In this paper, we will generalize the factorization results and the algorithm of [3] from motion polynomials to dual quaternion polynomials which no longer have to fulfill Study’s condition (Sects. 3.1–3.3). An obvious necessary condition for a factorization to exist is factorizability of the norm polynomial over the dual numbers. Moreover, we adopt the genericity assumption of [3] and generally require that the primal part has no real polynomial factor of positive degree.

The factorization results in [3] are proven for generic rational motions in this sense and it is known that their trajectories (orbits of points) are “entirely circular”, i.e. all intersection points with the plane at infinity have norm zero [6]. We will show that this no longer needs to be true for non-motion polynomials. Nonetheless, we prove a necessary factorizability condition that is related to the intersection points of trajectories with the plane at infinity and their norms (Sect. 3.4).

It is well-known that also non-motion polynomials can be used to parametrize rational motions (cf. for example [9–11]). In this more general setting, linear polynomials no longer parametrize just rotations or translations, they parametrize “vertical Darboux motions”, cf. [1], p. 321] and [11]. These are rotations around a fixed axis coupled with a harmonic oscillation along this axis. Rotations and translations are special cases and correspond to amplitude zero or infinity, respectively, of the harmonic oscillation. This allows us to construct mechanisms consisting of revolute, prismatic and “vertical Darboux joints”, which we show in Sect. 4.

The caveat of this approach is that no convenient mechanical realizations are known for vertical Darboux joints. One way to circumvent this problem is to use cylindrical joints that allow for an independent rotation around and translation along a fixed axis. They can replace vertical Darboux joints provided the mechanism still remains sufficiently constrained. In Sects. 4.1 and 4.2, we illustrate this at hand of quadratic polynomials where the mechanism construction via factorization into *motion* polynomials fails. Our results yield mechanisms consisting of two revolute and two cylindrical joints. We extend this construction to certain quadratic dual quaternion polynomials that do not satisfy Study’s condition.

## Preliminaries

Let us define the commutative, unital ring ${\mathbb {D}}={\mathbb {R}}[\varepsilon ]/\langle \varepsilon ^{2} \rangle$ of *dual numbers*. Elements of ${\mathbb {D}}$ can be written as $a+\varepsilon b$ with *a*, $b\in {\mathbb {R}}$. If $a\ne 0$, the dual number is invertible and its inverse is given by $(a-\varepsilon b)a^{-2}$. The ${\mathbb {D}}$-algebra generated by the base elements 1, $\mathbf {i}$, $\mathbf {j}$ and $\mathbf {k}$ is called the algebra of *dual quaternions*. The non-commutative multiplication of dual quaternions abides by the rules

$$\begin{aligned} \mathbf {i}^{2}=\mathbf {j}^{2}=\mathbf {k}^{2}=\mathbf {i}\mathbf {j}\mathbf {k}=-1,\qquad \mathbf {i}\varepsilon =\varepsilon \mathbf {i},\qquad \mathbf {j}\varepsilon =\varepsilon \mathbf {j},\qquad \mathbf {k}\varepsilon =\varepsilon \mathbf {k}. \end{aligned}$$

The *dual quaternion conjugate* of $q=q_{0}+q_{1}\mathbf {i}+q_{2}\mathbf {j}+q_{3}\mathbf {k}$ is given by ${q}^{*}=q_{0}-q_{1}\mathbf {i}-q_{2}\mathbf {j}-q_{3}\mathbf {k}$. Further we will call the dual number $\Vert q\Vert =q{q}^{*}={q}^{*}q$ the *dual quaternion norm* of *q* (despite the fact that this is not a norm in the usual sense). A dual quaternion can always be written as $q=p+\varepsilon d$, where *p* and *d* are elements of the real associative algebra ${\mathbb {H}}$ of (Hamiltionian) quaternions generated by $(1,\mathbf {i},\mathbf {j},\mathbf {k})$. We will call *p* the primal part and *d* the dual part of *q*. The set of invertible elements of ${\mathbb {DH}}$ will be denoted by ${\mathbb {DH}}^{\times }$. Its elements are all of the form $q=p+\varepsilon d$ with $\Vert p\Vert \ne 0$. In this case $\Vert q\Vert$ is invertible as well and we have $q^{-1}={q}^{*}\Vert q\Vert ^{-1}$.

It is well known, that the set of dual quaternions with real norm modulo real scalars is isomorphic to the group ${\text {SE}}(3)$ of rigid body displacements in three-space [12]. A dual quaternion $q=p+\varepsilon d$ has the norm $\Vert q\Vert =\Vert p\Vert +\varepsilon (p{d}^{*}+d{p}^{*})$ which is real precisely if $p{d}^{*}+d{p}^{*}=0$. This is called *Study’s condition*. We can think of this condition in the following way: the map $(p,d)\mapsto 1/2(p{d}^{*}+d{p}^{*})$ is a symmetric bilinear form on ${\mathbb {H}}^{2}$. The dual quaternion $q=p+\varepsilon d$ fulfills Study’s condition if and only if the quaternions *p* and *d* are orthogonal with respect to this bilinear form.

Dual quaternions $q=p+\varepsilon d$ with $p\ne 0$ fulfilling Study’s condition act on a point $(x_{1},x_{2},x_{3})\in {\mathbb {R}}^{3}$ by dual quaternion multiplication via

1 $$\begin{aligned} x\mapsto \frac{1}{\Vert p\Vert }(p-\varepsilon d)x({p}^{*}+\varepsilon {d}^{*})=\frac{1}{\Vert p\Vert }\left( px{p}^{*} +\varepsilon (p{d}^{*}-d{p}^{*})\right) , \end{aligned}$$

where $x=1+\varepsilon (x_{1}\mathbf {i}+x_{2}\mathbf {j}+x_{3}\mathbf {k})$. The first part of this map, namely $x\mapsto px{p}^{*}/\Vert p\Vert$ is a rotation given by the quaternion *p* while the second part adds the translation vector $(p{d}^{*}-d{p}^{*})/\Vert p\Vert$. For fixed *p*, the map $d\mapsto p{d}^{*}-d{p}^{*}$ is surjective onto the three-space spanned by $\mathbf {i}$, $\mathbf {j}$ and $\mathbf {k}$ and its kernel is spanned by *p*. Restricting the domain of this map to the orthogonal complement of *p*, i.e. to all *d* such that $p+\varepsilon d$ fulfills Study’s condition, we obtain a vector-space isomorphism. This shows that the group of dual quaternions which fulfill Study’s condition modulo the real multiplicative group ${\mathbb {R}}^\times$ is isomorphic to ${\text {SE}}(3)$.

The map in (1) however is well defined for all invertible dual quaternions, thus it can be extended to a surjective homomorphism between ${\mathbb {DH}}^\times$ and ${\text {SE}}(3)$. Two dual quaternions *q* and *h* represent the same rigid body displacement if and only if there exists a dual number $a+\varepsilon b$ with $a\ne 0$ such that $q=(a+\varepsilon b)h$ [9–11]. In fact, for every dual quaternion $q=p+\varepsilon d$ with $p\ne 0$, there exists a unique (up to real scalars) dual quaternion, which represents the same displacement and fulfills Study’s condition. It can be computed by multiplying the polynomial with the inverse of its norm polynomial (cf. [8]):

2 $$\begin{aligned} \left( \Vert p\Vert -\frac{\varepsilon }{2}(p{d}^{*}+d{p}^{*})\right) (p+\varepsilon d) = \Vert p\Vert p+\frac{\varepsilon }{2} (d{p}^{*}-p{d}^{*})p. \end{aligned}$$

### Rational Motions and Dual Quaternion Polynomials

One-parametric rigid body motions are maps $U\rightarrow {\text {SE}}(3)$ from an interval $U \subseteq {\mathbb {R}}$ into the group of rigid body displacements ${\text {SE}}(3)$. We may think of them as curves in ${\text {SE}}(3)$. A motion is called *rational,* if all trajectories are rational curves in ${\mathbb {R}}^{3}$. Rational motions can be represented by polynomials with dual quaternion coefficients fulfilling Study’s condition [5].

We consider polynomials $\sum _{i=0}^{n} m_it^i$ with dual quaternion coefficients $m_{0}$, $m_{1}$, $\ldots$, $m_n \in {\mathbb {DH}}$. The non-commutative multiplication of polynomials of this type is defined by the convention that the indeterminate *t* commutes with all coefficients. This turns the set of polynomials over the dual quaternions into a ring which we denote by ${\mathbb {DH}}[t]$.

Similar to the notions above we can define the conjugate of a dual quaternion polynomial $M = \sum _{i=0}^nm_it^i$ as ${M}^{*}=\sum _{i=0}^{n}{m_{i}}^{*}t^i$ and the norm polynomial as $\Vert M\Vert =M{M}^{*}={M}^{*}M$. It is a polynomial with coefficients in the dual numbers ${\mathbb {D}}$. Since dual quaternion multiplication is non-commutative, polynomials in ${\mathbb {DH}}[t]$ do not come with a canonical evaluation. We will use the so called *right evaluation M(h)* of the polynomial *M* at $h\in {\mathbb {DH}}$. It is obtained by writing the powers of the indeterminate on the right side of the coefficients before substituting *h* for *t*, i.e. $M(h) := \sum _{i=0}^nm_ih^i$. Dual quaternions for which the right evaluation of a polynomial *M* equals zero are called *right zeros* of *M*. The notions of *left evaluation* and *left zeros* can be defined by writing the indeterminate at the left side of the coefficients before substituting. We will occasionally refer to right zeros of *M* simply as “zeros”. A polynomial $M\in {\mathbb {DH}}[t]$ can always be written as $P+\varepsilon D$ where *P* and *D* are both polynomials with (Hamiltonian) quaternion coefficients. Again we call *P* the primal part and *D* the dual part of *M*.

All dual quaternion polynomials with the property that their norm polynomial does not lie in $\varepsilon {\mathbb {R}}[t]$ parameterize rational motions [9–11] via (1):

$$\begin{aligned} x\mapsto \frac{1}{\Vert P(t)\Vert }(P(t)-\varepsilon D(t))x({P(t)}^{*}+\varepsilon {D(t)}^{*}),\quad t \in {\mathbb {R}}. \end{aligned}$$

If dual quaternion polynomials are the same up to a real polynomial factor, they represent the same motions (this is even true for dual polynomial factors). Dual quaternion polynomials without a real polynomial factor are called *reduced*, the maximal real polynomial factor of $M\in {\mathbb {DH}}[t]$ will be denoted by ${\text {mrpf}}(M)$.

Dual quaternion polynomials with invertible leading coefficient and norm polynomial in ${\mathbb {R}}[t]$ are called *motion polynomials* [3, 7].

### The Vertical Darboux Motion

The simplest rational motions are rotations around a fixed axis and translations in a fixed direction. Both can be represented by linear motion polynomials, i.e. dual quaternion polynomials of degree one which fulfill Study’s condition. Given a point $(v_{1},v_{2},v_{3})\in {\mathbb {R}}^{3}$, the polynomial $t+v\in {\mathbb {DH}}[t]$ with $v=v_{1}\mathbf {i}+v_{2}\mathbf {j}+v_{3}\mathbf {k}$ parametrizes a rotation around the axis with direction *v*. Provided $\Vert (v_{1},v_{2},v_{3})\Vert =1$, the rotation angle is given by $2\cot ^{-1}(t)$. The polynomial $t+\varepsilon v$ parametrizes a translation by $-2v/t$. The rotation around an axis parallel to $(v_{1},v_{2},v_{3})$ and through the point $(x_{1},x_{2},x_{3})$ is given by

3 $$\begin{aligned} (1-\varepsilon x/2)(t+v)(1+\varepsilon x/2)=t+v+\varepsilon /2(vx-xv), \end{aligned}$$

where $$v = v_{1}\mathbf {i}+ v_{2}\mathbf {j}+ v_{3}\mathbf {k}$$ and $$x=x_{1}\mathbf {i}+ x_{2}\mathbf {j}+ x_{3}\mathbf {k}$$.

**Fig. 1:**
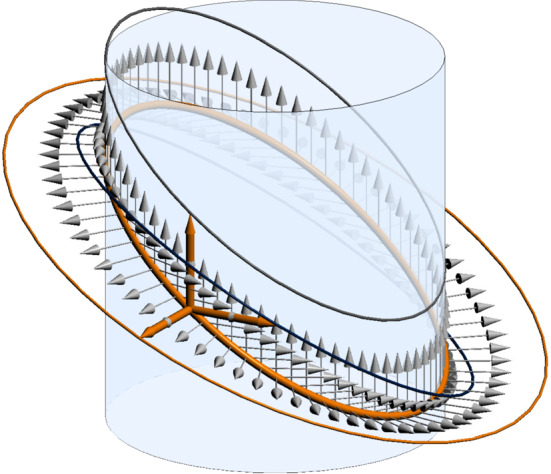
Vertical Darboux motion

But not all linear polynomials in ${\mathbb {DH}}[t]$ describe rotations or translations: In general, linear non-motion polynomials describe *vertical Darboux motions* which consist of a rotation around a fixed axis coupled with a harmonic oscillation along the same axis such that one period of oscillation corresponds to one full rotation (cf. [1], p. 321] for general information on vertical Darboux motions; the statement about their relation to linear polynomials can be found for example in [11]). We may view rotations and translations as special cases of vertical Darboux motions: They correspond to zero and infinite oscillation amplitude, respectively. General (not necessarily vertical) Darboux motions have the property that all trajectories are ellipses (or line segments). For vertical Darboux motions, these ellipses lie on cylinders around a fixed axis, the axis of the motion (Fig. 1), hence they are called *vertical* (German “aufrecht”).

A linear (non-motion) polynomial representing a vertical Darboux motion is for example $t+\mathbf {k}+\varepsilon$. Using (2) we can find the motion polynomial $(t^{2}+1)(t+\mathbf {k})+\varepsilon (1-t\mathbf {k})$ which represents the same vertical Darboux motion. This polynomial however is of degree three. Thus, by not restricting ourselves to motion polynomials, we can represent certain motions by polynomials with lower degree. In this view, neglecting Study’s condition introduces a new class of elementary motions, namely vertical Darboux motions, as they can be represented by linear non-motion polynomials.

## Factorization of Generic Dual Quaternion Polynomials

Factorization of dual quaternion polynomials into linear factors was the topic of [3, 7]. One of their intentions was to use factorization for the construction of mechanisms, where a linear factor corresponds to a revolute or prismatic (translational) joint [4]. The used factorization algorithms fundamentally rely on the fact that the dual quaternion polynomial $M = P + \varepsilon D$ is a *motion polynomial,* that is, it satisfies the Study condition $P{D}^{*} + D{P}^{*} = 0$.

In this article, we study the factorization theory of general dual quaternion polynomials which is interesting in its own right and provides us with additional possibilities for the construction of mechanisms. The factorization into linear factors corresponds to a decomposition of the motion into the product of vertical Darboux motions, coupled by the common parameter *t*, and with rotations and translations as special cases. Our aim is the generalization of the results from [3] to generic polynomials $M \in {\mathbb {DH}}[t]$.

### Fundamental Results

In this section we recall known results of [3] and also present slightly more general versions of Lemma 3 and Theorem 1 from [3]. They are generalized to dual quaternion polynomials which no longer have to fulfill Study’s condition. We give proofs for all new results, even if they are sometimes very similar to the proofs given in [3].

The first lemma concerns polynomial division in a dual quaternion setting. (We will only use this type of polynomial division in this paper and hence refrain from using the more explicit term “polynomial right division”.)

#### Lemma 3.1

([3], Lemma 3.1]) Let *A* and $B\in {\mathbb {DH}}[t]$ such that *B* is monic, i.e. its leading coefficient is 1. Then there exist unique *Q*, $R\in {\mathbb {DH}}[t]$ such that $A=QB+R$ and $\deg (R)<\deg (B)$. Further, if $h\in {\mathbb {DH}}$ is a root of *B*, then $A(h)=R(h)$.

Note that this statement can be generalized to a divisor *B* with invertible leading coefficient.

The next lemma states a well-known relation between right zeros and linear right factors which is also valid for polynomials over more general rings.

#### Lemma 3.2

([3], Lemma 3.2]) Let $M\in {\mathbb {DH}}[t]$. A dual quaternion $h\in {\mathbb {DH}}$ is a zero of *M* if and only if $t-h\in {\mathbb {DH}}[t]$ is a right factor of *M*.

If a polynomial $M = P + \varepsilon D$ satisfies Study’s condition, its norm polynomial $\Vert M\Vert$ is real. Linear factors of *M* are closely related to quadratic real factors of $\Vert M\Vert$. In our setting, the norm polynomial $\Vert M\Vert$ has dual numbers as coefficients. Its quadratic factors over the dual numbers are also important for computing linear factors of *M*. This manifests in the following lemma. Its proof follows the proof of [3], Lemma 3.3] but the more general assumptions require to consider a few more details.

#### Lemma 3.3

Let $M=P+\varepsilon D\in {\mathbb {DH}}[t]$ be a monic polynomial and $N\in {\mathbb {D}}[t]$ a monic polynomial of degree two which is a factor of $\Vert M\Vert$ whose primal part does not divide *P*. Then there exists a unique $h\in {\mathbb {DH}}$ such that $t-h\in {\mathbb {DH}}[t]$ is a right factor of *M* and $\Vert t-h\Vert =N$.

#### Proof

By Lemma 3.1 there exist *Q*, $R=r_{1}t+r_{0}\in {\mathbb {DH}}[t]$ such that $M=QN+R$. Using this we can see that the norm of *M* is of the following form:

$$\begin{aligned} \Vert M\Vert =(N\Vert Q\Vert +R{Q}^{*}+Q{R}^{*})N + \Vert R\Vert . \end{aligned}$$

The primal part of *N* does not divide *P*, therefore the primal part of *R* cannot be zero. Further, *N* must be a factor of $\Vert R\Vert$ since it is a factor of $\Vert M\Vert$. Thus there exists $\lambda \in {\mathbb {D}}$ such that $\Vert R\Vert =\lambda N$. The scalar $\lambda$ is even invertible as otherwise the primal part of *R* would vanish. The leading coefficient of $\Vert R\Vert$ is $\Vert r_{1}\Vert$, which has a non-zero primal part due to the facts that *N* is monic and $\lambda$ is invertible. This shows that $r_{1}$ is invertible and consequently $h:=r_{1}^{-1}r_{0}$ is the unique zero of *R*. By Lemma 3.2, there exists $\widetilde{r}\in {\mathbb {DH}}$ such that $R=\widetilde{r}(t-h)$ and further $\lambda N=\Vert R\Vert =(t-{h}^{*})\Vert \widetilde{r}\Vert (t-h)$. Since $\lambda$ is a dual number, $t-h$ is also a right factor of *N* and consequently of *M*. Monicity of *N* implies $N = \Vert t-h\Vert$. This shows existence.

To prove uniqueness let us assume there exists $\widetilde{h}$ such that $M(\widetilde{h})=N(\widetilde{h})=0$. Then $R(\widetilde{h})=0$, but the zero of *R* is unique. $\square$

We will see below that factorizability of $M=P+\varepsilon D$ implies that the norm polynomial $\Vert M\Vert$ must factor into quadratic polynomial factors over the dual numbers. Now we have all the tools to prove that this condition is also sufficient, provided *P* does not have a real polynomial factor of positive degree. This distinguishes factorization of general polynomials $M \in {\mathbb {DH}}[t]$ from the factorization of motion polynomials as investigated in [3, 7]: The latter unconditionally factorize if the primal part is free from non-trivial real factors since their norm polynomial always factors into quadratic polynomials.

Let us start by investigating the factorizability of polynomials in ${\mathbb {D}}[t]$.

#### Lemma 3.4

Let $p = f+\varepsilon g\in {\mathbb {D}}[t]$ be a monic polynomial. Further let us decompose the primal part of *p* into $f=\prod _{i=1}^mN_{i}^{n_{i}}$ where $N_{1}$, $\ldots$, $N_m\in {\mathbb {R}}[t]$ are coprime irreducible monic polynomials and $n_{1}$, $\ldots$, $n_m\in {\mathbb {N}}$ are positive integers. The polynomial *p* admits a factorization such that all factors are monic and have an irreducible primal part if and only if $\prod _{i=1}^mN_{i}^{n_{i}-1}$ is a factor of *g*.

#### Proof

Let us first assume that *p* admits a factorization $\prod _{j=1}^{n} (f_{j}+\varepsilon g_{j})$ where the $f_{j}$ are monic and irreducible (not necessarily coprime) factors of *f*. Obviously, we get $f=\prod _{j=1}^{n} f_{j}$ and further

$$\begin{aligned} f+\varepsilon g=f+\varepsilon \sum _{i=1}^ng_{i}\prod _{j\ne i}f_{j}, \end{aligned}$$

which shows that $\prod _{i=1}^mN_{i}^{n_{i}-1}$ is a factor of *g*.

Let us assume on the other hand that $\prod _{i=1}^mN_{i}^{n_{i}-1}$ is a factor of *g*. Then $p=\prod _{i=1}^mN_{i}^{n_{i}-1}\widetilde{p}$ where $\widetilde{p}=\prod _{i=1}^{m} N_{i} + \varepsilon \lambda$ is a monic polynomial, i.e. $\deg (\lambda )\le \deg (\widetilde{p}) -1$. Set $B := \prod _{i=1}^{m} N_{i}$ and, for $i \in \{1,$...$,m\}$, $B_{i} := \prod _{j\ne i}N_{j}$. The polynomial $\widetilde{p}$ admits a factorization of postulated shape, if there exist $\lambda _{i}\in {\mathbb {R}}[t]$ with $\deg (\lambda _{i})<\deg (N_{i})$ for $i=1$, $\ldots$, *m* such that

$$\begin{aligned} \widetilde{p}=\prod _{i=1}^{m} (N_{i}+\varepsilon \lambda _{i})=B+\varepsilon \sum _{i=1}^{m}\lambda _iB_{i}. \end{aligned}$$

Without loss of generality we may assume that there is $k\in {\mathbb {N}}$ such that $N_{1}$, $\ldots$, $N_k$ are quadratic polynomials, and $N_{k+1}$, $\ldots$, $N_m$ are linear. To show the existence of such $\lambda _{i}$, it is sufficient to show that the set of polynomials $B_{i}$, for $i=1$, $\ldots$, *m* and $tB_{i}$ for $i=1$, $\ldots$, *k* form an ${\mathbb {R}}$-basis of the real vector space of polynomials up to degree $\deg (\widetilde{p})-1=m+k-1$. Since the cardinality of this set is $m+k$, we only need to show linear independence. Let us take $\mu _{1},$...$,\mu _m\in {\mathbb {R}}$ and $\nu _{1},$...$,\nu _k\in {\mathbb {R}}$ such that

4 $$\begin{aligned} \sum _{i=1}^{m} \mu _iB_{i} + \sum _{i=1}^k \nu _itB_{i}=0. \end{aligned}$$

Since all $N_{i}$ are coprime, they have distinct complex roots $z_{i}$, $\overline{z_{i}}\in \mathbb {C}\backslash {\mathbb {R}}$ for $i=1$, $\ldots$, *k* and $z_{i}\in {\mathbb {R}}$ for $i=k+1$,$\ldots$, *m*. The polynomials $B_{i} = \prod _{j\ne i}^{m} N_{j}$ evaluated at $z_\ell$ or $\overline{z_\ell }$ are all zero, except for $i=\ell$. Thus, evaluating the left hand side of (4) at these zeros results in a system of equations

5 $$\begin{aligned} \begin{aligned} \mu _{i} + \nu _iz_{i}&=0,\\ \mu _{i} + \nu _{i}\overline{z_{i}}&=0, \end{aligned} \end{aligned}$$

for $i=1, \ldots ,k$ and $\mu _{i}=0$ for $i > k$. The difference of these two equations yields

$$\begin{aligned} 2\nu _{i}{\text {Im}}{z_{i}} = 0. \end{aligned}$$

Since $z_{i} \in {\mathbb {C}}\setminus {\mathbb {R}}$, ${\text {Im}}{z_{i}} \ne 0$ so that $\nu _{i} = 0$ and hence also $\mu _{i} = 0$ follows. Thus, the set of Eq. (5) only has the solution $\mu _{i}=\nu _{i}=0$ for $i=1,$..., *k*. Therefore, the polynomials $B_{i}$ and $tB_{i}$ are linearly independent and *p* consequently admits a factorization of the desired shape. $\square$

#### Theorem 3.5

Let $M=P+\varepsilon D\in {\mathbb {DH}}[t]$ be a polynomial with no real polynomial factor in the primal part (${\text {mrpf}}(P)=1$) and $\Vert P\Vert =\prod _{i=1}^{m} N_{i}^{n_{i}}$ for irreducible, quadratic and coprime real polynomials $N_{1},\ldots ,N_m \in {\mathbb {R}}[t]$ and positive integers $n_{1},$...$,n_m \in {\mathbb {N}}$. The polynomial *M* admits a factorization if and only if $\prod _{i=1}^{m} N_{i}^{n_{i}-1}$ is a factor of $\Vert M\Vert$. (This is the case if and only if $\Vert M\Vert$ factorizes in the sense of Lemma 3.4).

#### Proof

Let us assume $M=\prod _{j=1}^k F_{j}$, where $F_{j}=P_{j}+\varepsilon D_{j} \in {\mathbb {DH}}[t]$ are linear polynomials. Then

$$\begin{aligned} \Vert M\Vert =\prod _{j=1}^k \Vert F_{j}\Vert = \prod _{i=1}^mN_{i}^{n_{i}} + \varepsilon \sum _{j=1}^k (P_{j}{D_{j}}^{*}+D_{j}{P_{j}}^{*}) \Vert P_{j}\Vert ^{-1} \prod _{i=1}^{m} N_{i}^{n_{i}}. \end{aligned}$$

Since for every $j=1,\ldots ,k$ there exists $i\in \{1,\ldots ,m\}$ such that $\Vert P_{j}\Vert = N_{i}$, we can conclude that $\prod _{i=1}^{m} N_{i}^{n_{i}-1}$ is a factor of $\Vert M\Vert$.

Let us assume on the other hand that $$\prod _{i=1}^{m} N_{i}^{n_{i}-1}$$ is a factor of $$\Vert M\Vert $$. By Lemma 3.4 we know that $$\Vert M\Vert $$ admits a factorization into quadratic and monic polynomials $$q_{1}$$, $$\ldots $$, $$q_k$$ in $${\mathbb {D}}[t]$$. By assumption *P* does not have a real factor. Thus we can use Lemma 3.3 to find $$h_k\in {\mathbb {DH}}$$ and $$Q\in {\mathbb {DH}}[t]$$ such that $$M= Q(t-h_k)$$ and $$\Vert t-h_{k}\Vert =q_k$$. Obviously, *Q* cannot have a real polynomial factor in the primal part as otherwise *M* would have a factor in the primal part which contradicts our assumptions. Further $$\Vert M\Vert =\Vert Q\Vert q_k$$, whence $$\Vert Q\Vert =\prod _{i=1}^{k-1}q_{i}$$. Thus we can use Lemma 3.3 recursively to obtain $$h_{1}, $$...$$, h_{k-1}\in {\mathbb {DH}}[t]$$ such that $$M=\prod _{i=1}^k (t-h_{i})$$ and $$\Vert t-h_{i}\Vert =q_{i}$$. $$\square $$

#### Example 1

The polynomial $M=P+\varepsilon D$ with $P=(t-\mathbf {i})(t-\mathbf {k})$ and $D=t-\mathbf {j}$ does not admit a factorization. The norm of *M* equals $(t^{2}+1)^{2}+2\varepsilon (t^{3}+1)$ but its dual part does not have the factor $t^{2}+1$. This violates the necessary factorization condition of Theorem 3.5.

### Algorithm

The proofs of Lemma 3.3 and Theorem 3.5 are constructive and similar to the proofs in [3]. The difference is, that we need to use quadratic *dual* polynomial factors of the norm polynomial. Nonetheless the same Algorithm 1 can be used to compute factorizations.

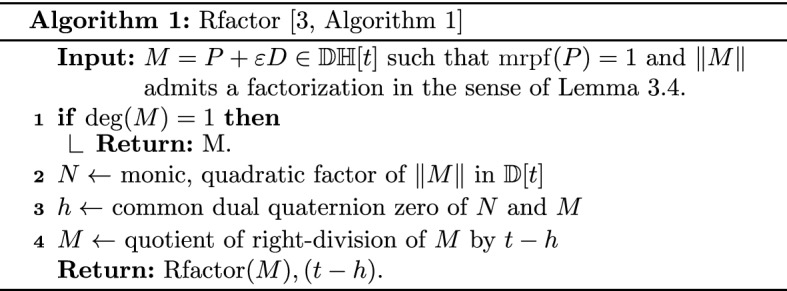



Algorithm 1 is not deterministic as its output depends on the choice of the quadratic factor *N* in Line 2. In the generic case, the norm polynomial of *P* has *n* coprime irreducible quadratic factors in ${\mathbb {R}}[t]$. Then the factorization of $\Vert M\Vert$ is unique up to permutations of the factors, as we have seen in the proof of Lemma 3.4. In this case, there exist *n*! different factorizations of *M*, as in the original case of “generic motion polynomials” [3]. The situation is different, if the norm of *P* has quadratic factors of higher multiplicity.

#### Corollary 3.6

Let $M=P+\varepsilon D\in {\mathbb {DH}}[t]$ be a dual quaternion polynomial that admits a factorization. If the norm of *P* has a quadratic factor $N \in {\mathbb {R}}[t]$ with multiplicity $m \ge 2$, then *M* admits infinitely many factorizations.

#### Proof

The norm polynomial $\Vert M\Vert$ has the factor $N^{m}+\varepsilon N^{m-1}\lambda$ for a linear polynomial $\lambda \in {\mathbb {R}}[t]$. There are infinitely many ways to write $\lambda$ as a sum of *m* linear polynomials $\lambda _{i}$ so that

$$\begin{aligned} N^{m}+\varepsilon N^{m-1}\lambda = N^{m}+\varepsilon N^{m-1}\sum _{i=1}^{m} \lambda _{i} = \prod _{i=1}^{m} (N+\varepsilon \lambda _{i}). \end{aligned}$$

Consequently, $\Vert M\Vert$ has infinitely many factorizations and so does *M*. $\square$

### Translational Factors

Algorithm 1 is known to work for many more general cases (without the assumption ${\text {mrpf}}(P) = 1$) but cases of failure are known as well. In particular, it was already observed in [3] that the algorithm is applicable to motion polynomials where the irreducible real polynomial factors of its primal part are linear and at most of multiplicity one. This is still the case for general polynomials $M=cP+\varepsilon D\in {\mathbb {DH}}[t]$, where $c=\prod _{i=1}^{n} c_{i}\in {\mathbb {R}}[t]$ is a product of distinct linear polynomials $c_{i}$ and *P* has no real polynomial factor. The norm polynomial of *M* has *c* as factor and the primal part of $\Vert M\Vert$ has $c^{2}$ as factor. Thus we can use Lemma 3.4 to find linear $\lambda _{i}\in {\mathbb {R}}[t]$ such that $\Vert M\Vert$ has the factors $c_{i}^{2}+\varepsilon \lambda _{i}$. The primal part of these factors do not divide the primal part of *M* due to the assumption that every linear factor of the primal part has multiplicity one, thus we can use Lemma 3.3 to compute linear factors $c_{i} + \varepsilon (d_{1}\mathbf {i}+ d_{2}\mathbf {j}+ d_{3}\mathbf {k})$ of *M* and consequently, the Algorithm 1 still works in this more general setting. The linear factors $c_{i} + \varepsilon (d_{1}\mathbf {i}+ d_{2}\mathbf {j}+ d_{3}\mathbf {k})$ parameterize all translations in the fixed direction $(d_{1},d_{2},d_{3})$, hence the name “translational factors”.

If on the other hand, we have a reduced polynomial $M=c^{2}P+\varepsilon D$ where $c\in {\mathbb {R}}[t]$ is a linear polynomial, it does not admit a factorization. To see this, let us assume the contrary $M=\prod _{i=1}^{n} (P_{i}+\varepsilon D_{i})$. Since the primal part of *M* is the product of the primal parts of the factors, we know that two of the factors have *c* as primal part. Thus, there exist $Q_{1}$, $Q_{2}$, $F_{1}$ and $F_{2}\in {\mathbb {H}}[t]$ such that $M=(cQ_{1}+\varepsilon F_{1})(cQ_{2}+\varepsilon F_{2}) = c(cQ_{1}Q_{2} + \varepsilon (Q_{1}F_{2}+F_{1}Q_{2}))$. But this contradicts the assumption that *M* is reduced.

### Circularity and Factorizability

It is known that generic motion polynomials $Q = P + \varepsilon D$ (polynomials in ${\mathbb {DH}}[t]$ with ${\text {mrpf}}(P) = 1$ and $P{D}^{*} + D{P}^{*} = 0$) have the following properties:

- Their norm polynomial $\Vert P\Vert$ factors into quadratic irreducible polynomials over ${\mathbb {R}}$,
- they always admit factorizations and
- their generic trajectories are entirely circular [6, Theorem 1].

The circularity of an algebraic curve is defined as half the number of intersection points with the absolute circle at infinity counted with their multiplicities. A curve is entirely circular if all its intersections with the plane at infinity lie on the absolute circle.

Let us quickly cast these concepts into algebraic equations. In the dual quaternion formalism, ${\mathbb {P}}^{3}({\mathbb {R}})$ is identified with the projective space over the vector space spanned by 1, $\varepsilon \mathbf {i}$, $\varepsilon \mathbf {j}$, $\varepsilon \mathbf {k}$. Writing a general vector as $x_{0} + \varepsilon x$ with $x = x_{1}\mathbf {i}+ x_{2}\mathbf {j}+ x_{3}\mathbf {k}$, the plane at infinity is described by $x_{0} = 0$ and the absolute circle at infinity is given by the additional equation $\Vert x\Vert = 0$. If $x_{0} + \varepsilon x$ is not a constant dual quaternion but a dual quaternion polynomial (that is, a rational parameteric curve), the parameter values of its intersection points with the plane at infinity are the zeros of $x_{0}$. If they are also zeros of $\Vert x\Vert$, they contribute, with their respective multiplicity, to the curve’s circularity.

Consider now a general polynomial $M = P + \varepsilon D$ with ${\text {mrpf}}(P) = 1$ whose norm polynomial $\Vert M\Vert$ factors over ${\mathbb {D}}$ into quadratic polynomials. We have already seen that *M* admits a factorization. Now we investigate necessary properties of its trajectories. It turns out that these are much weaker than in the motion polynomial case.

#### Theorem 3.7

Let $M=P+\varepsilon D\in {\mathbb {DH}}[t]$ be a polynomial such that ${\text {mrpf}}(P)=1$ and *M* admits a factorization. Then the trajectories of the motion parameterized by *M* have the following property: All intersection points of the curve with the plane at infinity with multiplicity $\mu >1$ intersect the absolute circle with multiplicity $\mu$.

#### Proof

The trajectory of an arbitrary point $x_{0}+\varepsilon x$ with $x=x_{1}\mathbf {i}+x_{2}\mathbf {j}+x_{3}\mathbf {k}$ in projective three-space with respect to *M* is given by

6 $$\begin{aligned} (P-\varepsilon D)(x_{0}+\varepsilon x)({P}^{*}+\varepsilon {D}^{*})= & {} x_{0}\Vert P\Vert +\varepsilon (Px{P}^{*}+x_{0}(P{D}^{*}-D{P}^{*})).\nonumber \\ \end{aligned}$$

This curve’s intersection points with the plane at infinity correspond to zeros of its primal part and consequently to the factors of $\Vert P\Vert$. Let us write $\Vert P\Vert =\prod _{i=1}^{m} N_{i}^{n_{i}}$ where $N_{1}, \ldots , N_m\in {\mathbb {R}}[t]$ are coprime irreducible polynomials. We need to show that all $N_{i}^{n_{i}}$ with $n_{i}>1$ are factors of the norm of the dual part in (6). Let us have a look at the polynomial

$$\begin{aligned} \Vert Px{P}^{*}+x_{0}(P{D}^{*}-D{P}^{*})\Vert= & {} \Vert P\Vert ^{2}\Vert x\Vert - x_{0}\Vert P\Vert (Px{D}^{*}+D{x}^{*}{P}^{*})\\&\quad + x_{0}P(x{P}^{*}D+{D}^{*}P{x}^{*}){P}^{*} +x_{0}^{2}\Vert P{D}^{*}-D{P}^{*}\Vert . \end{aligned}$$

The first two summands have $\Vert P\Vert$ as a factor. The middle factor of the third summand is a real polynomial, thus it commutes with *P* and therefore the third summand also has the factor $\Vert P\Vert$. For the last summand it holds

$$\begin{aligned} \Vert P{D}^{*}-D{P}^{*}\Vert&=4\Vert P\Vert \Vert D\Vert -(P{D}^{*}+D{P}^{*})^{2}. \end{aligned}$$

Since *M* admits a factorization, we know that $c := \prod _{i=1}^{m} N_{i}^{n_{i}-1}$ is a factor of the dual part of $\Vert M\Vert$ which is $P{D}^{*}+D{P}^{*}$. Thus the last summand has the factor $c^{2}$ which in turn has the factors $N_{i}^{n_{i}}$ with $n_{i}>1$. This proves the statement. $\square$

The converse of Theorem 3.7 is not true which can be seen in the following example.

#### Example 2

Let $M=(t-\mathbf {i})(t-\mathbf {k})+\varepsilon (t-\mathbf {j})$ be the polynomial of Example 1. The polynomial $Q=M(t-\mathbf {k})^{2}$ does not admit a factorization, because it does not fulfill the requirements of Theorem 3.5, but its norm polynomial $\Vert Q\Vert$ has the factor $(t^{2}+1)^{2}$. From the proof above we can conclude that the trajectories of *Q* are entirely circular even though the polynomial does not admit a factorization.

## Construction of Mechanisms

**Fig. 2:**
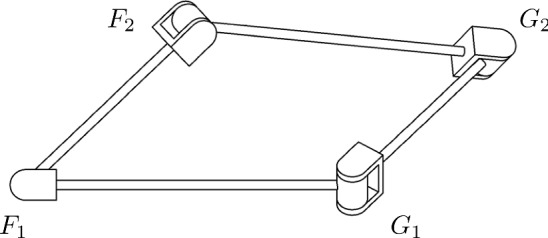
Realization of two factorizations $M = F_{1}F_{2} = G_{1}G_{2}$ as a mechanisms

In [3, 7] the authors presented algorithms to find factorizations of monic motion polynomials $M=P+\varepsilon D \in {\mathbb {DH}}[t]$ into linear factors where all factors are motion polynomials themselves, i. e. they parametrize rotations or translations. This composition of rotations and translations can be used to construct an open kinematic chain with revolute and prismatic joints, coupled by the common parameter *t*, which can perform the rational motion given by *M*. Different factorizations yield different open chains which can be coupled to reduce the degrees of freedom in the resulting kinematic structure. This is illustrated in Fig. 2 for a quadratic motion polynomial *M* with two distinct factorizations $M=F_{1}F_{2}=G_{1}G_{2}$. Each factorization yields a kinematic chain consisting of two revolute joints, which are interlocked to obtain the depicted mechanism. The connecting link of $F_{2}$ and $G_{2}$ performs the desired one-parametric motion given by *M*.

As is usual in mechanism science, we denote revolute joints by the letter “R” and prismatic joints by the letter “P”. The mechanism of Fig. 2 would then be referred to as closed RRRR- or 4R-mechanism.

In this paper we consider polynomials in ${\mathbb {DH}}[t]$ which no longer need to fulfill Study’s condition. This results in factorizations where not all linear factors are motion polynomials and hence represent vertical Darboux motions. In theory this would allow the construction of kinematic chains with “vertical Darboux” joints (and revolute or prismatic joints in special cases). Darboux joints are problematic from an engineering viewpoint. Thus we will focus on a different approach where vertical Darboux joints are simply replaced by cylindrical (“C”) joints that allow the simultaneous rotation around and translation along an axis. Of course, this comes with the downside that cylindrical joints have two degrees of freedom. This will increase the degrees of freedom of the corresponding open chain but, if it is sufficiently constrained otherwise, does not increase the degrees of freedom of the complete mechanical system: The cylindrical joints will actually just perform the respective vertical Darboux motions. In this way, kinematic chains obtained by the known algorithms might be coupled with chains obtained by our results to construct mechanisms with revolute, prismatic and cylindrical joints.

As cylindrical joints introduce an additional degree of freedom, it might be desirable to keep the number of C-joints low. This can be done by a certain extend by choosing appropriate quadratic factors of the norm polynomial in Algorithm 1. We will use this in Sect. 4.2 to obtain over-constrained mechanisms corresponding to certain quadratic dual quaternion polynomials which is only feasible by keeping the number of C-joints minimal.

### Bennett Motions and Four-Bar Linkage

Quadratic motion polynomials generically represent so-called Bennett motions [2]. In general, i.e. when the norm polynomial is the product of two coprime real quadratic polynomials, we can use the factorization algorithm for motion polynomials to obtain two distinct factorizations which in turn correspond to a closed chain with four revolute joints, a closed 4R-linkage or “Bennett mechanism” (Fig. 2). But if the norm polynomial is a quadratic irreducible polynomial to the power of two, there exists only one factorization into linear motion polynomials and the construction fails. (This is actually a quite common case. The motion can be obtained by composing two rotations around different axes but with identical angular velocities.) Our algorithm allows us to find another factorization into linear (non-motion) polynomials, which enables us to construct an RRCC-linkage that is capable of performing the given Bennett motion.

#### Example 3

The motion polynomial $M=t^{2}-((1+2\varepsilon )\mathbf {i}+\mathbf {k}+ \varepsilon \mathbf {j})t-\mathbf {j}+\varepsilon (\mathbf {i}-2)$ has the norm polynomial $(t^{2}+1)^{2}$. It only admits a unique factorization into motion polynomials, namely $M=G_{1}G_{2}$ with $G_{1}=t-\mathbf {i}-\varepsilon \mathbf {j}$ and $G_{2}=t-\mathbf {k}-2\varepsilon \mathbf {i}$. But we can also write $\Vert M\Vert =(t^{2}+1+\varepsilon \lambda )(t^{2}+1-\varepsilon \lambda )$ for an arbitrary linear polynomial $\lambda =\lambda _{1}t+\lambda _{0}\in {\mathbb {R}}[t]$. Using these factors of the norm polynomial in Algorithm 1 yields the additional factorization $M=F_{1}F_{2}$ where

$$\begin{aligned} F_{1}&= G_{1} + \frac{\varepsilon }{2}((\mathbf {i}+\mathbf {k})\lambda _{0}-(1+\mathbf {j})\lambda _{1}),\\ F_{2}&= G_{2} -\frac{\varepsilon }{2}((\mathbf {i}+\mathbf {k})\lambda _{0}-(1+\mathbf {j})\lambda _{1}). \end{aligned}$$

**Fig. 3:**
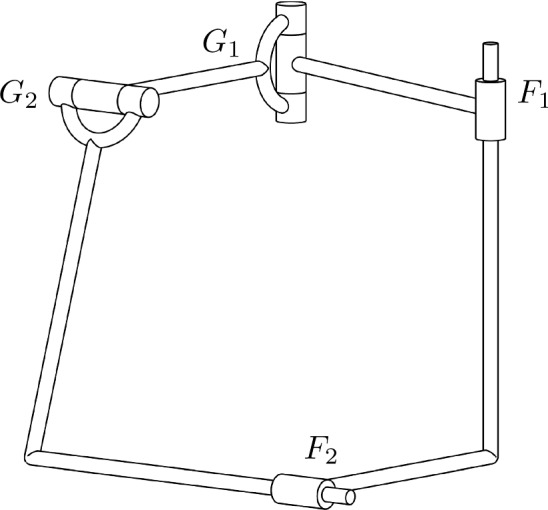
An RRCC-mechanism to perform a Bennett motion

Note that $F_{1}$ and $F_{2}$ are not motion polynomials and therefore parametrize “proper” vertical Darboux motions. Thus we need to use C-joints to obtain an open kinematic chain corresponding to this factorization. We can couple this 2C-chain with the 2R-chain obtained by the first factorization, since $M=F_{1}F_{2}=G_{1}G_{2}$. To specify the mechanism corresponding to these factorizations, it suffices to calculate the angles and distances between the joint axes. They are independent from the mechanism’s configuration which depends on the common joint parameter *t*. In the following, we will calculate these values for the mechanism corresponding to the values $\lambda _{0}=3$, $\lambda _{1}=4$. To find the axes of $G_{1}$ and $G_{2}$, we can use (3). They are $(0,0,-1)+{\mathbb {R}}(1,0,0)$ and $(0,-2,0)+{\mathbb {R}}(0,0,1)$. The axes of $F_{1}$ and $F_{2}$ can be found by computing the fixed line of these transformations. For $F_{1}$ we know the direction of the axis from the primal part, namely (1, 0, 0), thus it suffices to find a point on the axis. For $t=0$, the image of an arbitrary line $(x_{1},x_{2},x_{3})+{\mathbb {R}}(1,0,0)$ is $(x_{1}+4,-x_{2} - 3,-x_{3} - 6) + {\mathbb {R}}(1,0,0)$. Subtracting the point $(x_{1},x_{2},x_{3})$ should yield the line ${\mathbb {R}}(1,0,0)$. We infer $x_{2}=-3/2$, $x_{3}=-3$ while $x_{1}$ can be chosen arbitrarily, for example $x_{1}=0$. The axis of $F_{2}$ can be found similarly, it is given by $(-2,-7/2,0)+{\mathbb {R}}(0,0,1)$. The angles between $F_{1}$ and $F_{2}$, or $G_{1}$ and $G_{2}$ are $\pi /2$, respectively. The axes of $F_{1}$ and $G_{1}$ or $F_{2}$ and $G_{2}$ are parallel. The distance between $F_{1}$ and $F_{2}$ or $G_{1}$ and $G_{2}$ is 2, the distance between $F_{1}$, $G_{1}$ or $F_{2}$, $G_{2}$ is 5/2, respectively. Figure 3 depicts the resulting mechanism.

### Quadratic Polynomials and Four-Bar Linkages

While all rational motions can be represented by motion polynomials, the degree of this representation might not be optimal. Consider a generic polynomial $Q = P + \varepsilon D$. We can use equation (2) to obtain the motion polynomial $M=\Vert P\Vert P+\frac{1}{2}\varepsilon (P{D}^{*}-D{P}^{*})P$ which represents the same motion. In general, the degree of *M* is three times the degree of *Q* (it can be lower, if real polynomials can be canceled). Thus factorizing *Q* instead of *M* might result in fewer factors and therefore in mechanisms with fewer joints. We study this in the case of quadratic polynomials in ${\mathbb {DH}}[t]$. Their representations as motion polynomials are of degree four or six, which results in factorizations with four or six linear motion polynomials, respectively. If we get six linear factors, each factorization corresponds to an open 6R-chain which parametrize the whole ${\text {SE}}(3)$. Coupling different 6R-chains therefore does not constrain the obtained mechanism and we cannot obtain a mechanism with a degree of freedom lower than six. In the case that *M* has degree four, factorizations of *M* correspond to open 4R-chains. Interlocking two different 4R-chains yields a closed 8R-mechanism which has two degrees of freedom. To obtain a mechanism which only allows for a one-parametric motion we need to couple this closed 8R-mechanism with a third open 4R-chain. This approach therefore results in a rather complicated mechanism. The quadratic polynomial on the other hand admits a factorization into two linear polynomials, provided it meets the requirements of Theorem 3.5. This allows for the construction of open CC-chains. This is actually just a special RPRP-chain (revolute and prismatic joints alternate) in disguise and in this sense does not offer advantages over 4R-chains.

However, if the norm polynomial of the primal part is a square, there exist two different factorizations, each with a linear motion polynomial as one factor. Combining the corresponding kinematic chains, an open RC- and an open CR-chain, gives a closed RCRC four-bar linkage. It has just one degree of freedom and performs the motion given by *Q* and *M*.

#### Example 4

For $P=t^{2}-t(\mathbf {i}+\mathbf {k})-\mathbf {j}$ and $D=(2\mathbf {k}-4\mathbf {i}-1)t+(\mathbf {i}-\mathbf {j}-1)$ the motion polynomial $M=\Vert P\Vert P+\frac{1}{2}\varepsilon (P{D}^{*}-D{P}^{*})P$ is of degree four (after dividing off a real polynomial factor), but the representation as a general polynomial $Q = P + \varepsilon D$ is just of degree two. The motion polynomial *M* admits factorizations into four linear motion polynomials and thus the construction of open 4R-chains. The general polynomial *Q* on the other hand can be factored as $Q=F_{1}F_{2}=G_{1}G_{2}$ with

$$\begin{aligned} F_{1}= & {} t-\mathbf {i}+3\varepsilon \mathbf {k},\quad F_{2} = t-\mathbf {k}-e(1+4\mathbf {i}+\mathbf {k}),\\ G_{1}= & {} t-\mathbf {i}-\varepsilon (1+\mathbf {i}+\mathbf {j}-2\mathbf {k}),\quad G_{2} = t-\mathbf {k}-\varepsilon (3\mathbf {i}-\mathbf {j}). \end{aligned}$$

**Fig. 4:**
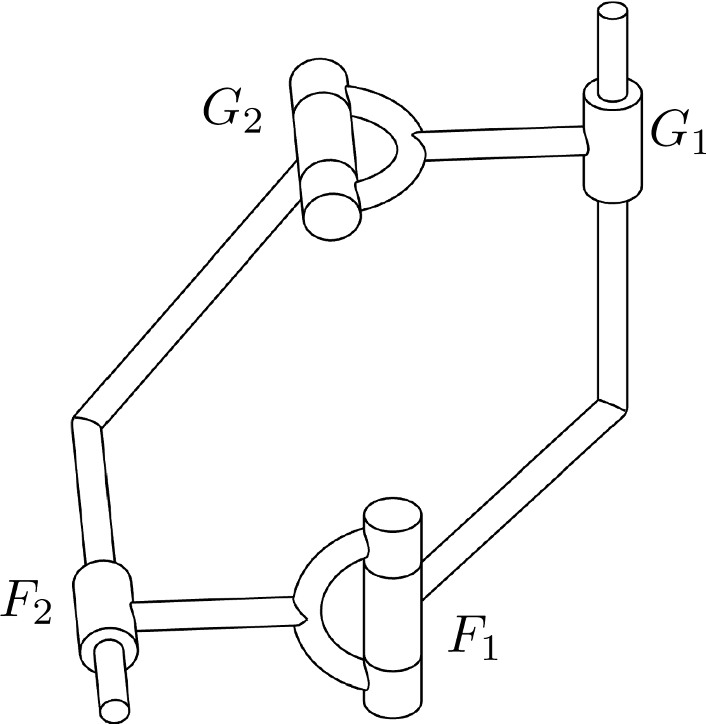
An RCRC-mechanism constructed from a quadratic polynomial

The first factorization $F_{1}F_{2}$ corresponds to an RC-chain, the second factorization $G_{1}G_{2}$ gives a CR-chain. Combining them yields the RCRC four-bar linkage of Fig. 4.

## Concluding Remarks

We have shown how to factorize polynomials over the dual quaternions under two assumptions:

- The norm polynomial factorizes into quadratic factors over the dual numbers and
- the primal part does not have a real polynomial factor of positive degree.

The first condition is obviously necessary for existence of a factorization, the second condition is a certain restriction. Precise criteria for existence of factorizations and algorithms for their computation in the excluded non-generic case are subject of ongoing research. Even in case of motion polynomials a complete answer is yet unknown.

We consider our factorization results as interesting in their own right but we also demonstrated that they complement well the known constructions of mechanisms from motion polynomials. It seems that abandoning Study’s condition (and thus giving up uniqueness of motion representation) is a promising concept and deserves further investigations.
